# A Qualitative Study of Alcohol, Health and Identities among UK Adults in Later Life

**DOI:** 10.1371/journal.pone.0071792

**Published:** 2013-08-07

**Authors:** Graeme B. Wilson, Eileen F. S. Kaner, Ann Crosland, Jonathan Ling, Karen McCabe, Catherine A. Haighton

**Affiliations:** 1 Institute of Health and Society, Newcastle University, Newcastle upon Tyne, United Kingdom; 2 Department of Pharmacy Health and Wellbeing, University of Sunderland, Sunderland, United Kingdom; The University of New South Wales, Australia

## Abstract

Increasing alcohol consumption among older individuals is a public health concern. Lay understandings of health risks and stigma around alcohol problems may explain why public health messages have not reduced rates of heavy drinking in this sector. A qualitative study aimed to elucidate older people's reasoning about drinking in later life and how this interacted with health concerns, in order to inform future, targeted, prevention in this group. In 2010 a diverse sample of older adults in North East England (ages 50–95) participated in interviews (n = 24, 12 male, 12 female) and three focus groups (participants n = 27, 6 male, 21 female). Data were analysed using grounded theory and discursive psychology methods. When talking about alcohol use older people oriented strongly towards opposed identities of normal or problematic drinker, defined by propriety rather than health considerations. Each of these identities could be applied in older people's accounts of either moderate or heavy drinking. Older adults portrayed drinking less alcohol as an appropriate response if one experienced impaired health. However continued heavy drinking was also presented as normal behaviour for someone experiencing relative wellbeing in later life, or if ill health was construed as unrelated to alcohol consumption. Older people displayed scepticism about health advice on alcohol when avoiding stigmatised identity as a drinker. Drinking patterns did not appear to be strongly defined by gender, although some gendered expectations of drinking were described. Identities offer a useful theoretical concept to explain the rises in heavy drinking among older populations, and can inform preventive approaches to tackle this. Interventions should engage and foster positive identities to sustain healthier drinking and encourage at the community level the identification of heavy drinking as neither healthy nor synonymous with dependence. Future research should test and assess such approaches.

## Introduction

Trends towards increased consumption of alcohol even at levels well short of dependence raise concerns for public health, particularly among those in later life. Alcohol consumption and alcohol-related deaths or problems have recently increased among older age groups in many developed countries, including the USA, Australia and many countries of the European Union [1]–[5]. The US National Institute on Alcohol Abuse and Alcoholism recommends that healthy adults aged over 65 years who do not take medications should not drink more than 3 drinks (42 g alcohol) per day or 7 drinks (92 g alcohol) per week [6]. Yet 10.8% of men and 2.9% of women in a large US sample aged over 65 reported exceeding these limits, compared with 28.6% of men and 10.3% of women in a UK sample the same age [7] and just over a fifth of samples of comparable age in Finland and Belgium [8]. In the USA, 23% of men and 9% of women aged 50–64, and 14% of men and 3% of women aged 65 and above, report binge drinking (consuming five or more drinks) on at least one occasion in the preceding month [9], while 27% of Europeans aged over 54 years report doing so on a weekly basis [10]. Frequent alcohol consumption can also have a negative impact on health, and is positively associated with age. In England, 28% of men over 65 years and 14% of women over 65 drink alcohol more than 5 times per week, while in Australia 15% of those aged over 65 report daily consumption compared with 4% of those aged under 50 [3], [11]. Other work has however found that frequency of consumption may decrease across the older age group [12], which suggests there is still much important research to be conducted in order to gain a detailed picture of alcohol consumption in older people.

Risk of mortality over 20 years is increased among individuals aged 55–65 years consuming a daily average of 3 standard drinks (42 g alcohol) [13]; in a sample of male general practitioners aged 48–74 years this risk was increased at an average daily consumption of 2 drinks or more [14]. Decreasing consumption below this level may not continue to reduce risk of mortality, however. Older adults whose average daily consumption fell between 1 and 2 standard drinks per day showed no increase in combined risk of disability and mortality compared with those consuming an average of 0 to 1 standard drinks per day [7]. Older people are more likely than younger adults to suffer from chronic health problems, bereavement or isolation and to use medications to a greater extent [15], [16]. These medications may interact with any alcohol consumed, for instance increasing their likelihood of falls or problems with the medication [12]. Encroaching health or social problems can lead to increased alcohol consumption either to self-medicate or to cope. Consuming more than 14 units of alcohol per week in this age group has been found to be positively associated with symptoms of depression and anxiety and less social support, though the direction of causality in this relationship is unclear [17]. For these reasons, regular and heavy drinking (in excess of recommended guidelines) can have a greater impact on physical and mental health in older individuals than in younger ones, and can have an effect at lower levels of consumption than would be the case in middle age or early adulthood [18]–[20].

Heavy drinking is more widespread than physiological or psychological dependence on alcohol, and is often targeted by national policy, for instance through taxation, public health messages about its impacts and support for preventive non-specialist approaches such as brief interventions [21]. Given these efforts, it is important to understand why older age groups are drinking more heavily than previously. Interventions to reduce alcohol consumption aim to encourage reasoned decisions about health [22]. However, responses to public health messages may be weak where the risk to health is from a consumable substance rather than an organism (such as influenza), where that substance can be perceived as desirable, and where health risks are deferred [23]; all qualities characteristic of alcohol consumption. In such circumstances, individuals weighing up the immediate and long-term consequences of a risky behaviour may, through engagement with cultural channels or humour, favour contradictory theories or findings around health risks, and also speculate on the motives or partiality of sources of advice [24]. This can build a rationale privileging the perceived benefits of heavy drinking of alcohol over generalised understanding of its risks to health [25]. Yet this may not fully explain increasing consumption in older people; the same considerations should apply to younger adults whose average drinking levels have reduced over recent years but who are more likely to defer health considerations to a later time of life [26], [27].

Rather than viewing health risks from alcohol as being outweighed by its desirability, older people may reason around alcohol primarily in social or moral rather than health terms [24]. Heavy drinking in this age group is subject to particular stigma [18], [28]–[30]. Given the gendered patterns of alcohol consumption [31], this may be especially so for women's drinking; women in mid to later life, for instance, may perceive particular pressure to present their drinking as appropriate and moderate in a way that men of the same age do not [32], [33]. Although stigma may increase the isolation older people already suffer disproportionately, and hamper the identification and prevention of risks to their health from alcohol, there has been little elaboration of how this is maintained and exerts influence. Identity theory suggests that over successive transitions in life, older people will have rehearsed and accumulated preferred versions of self within which to frame themselves and their experience [34], [35]. These identities will be multiple, mutable and responsive to personal, temporal, social and cultural contexts [36]. An older person may for instance move between representing themselves as a matriarch or patriarch, a patient, someone winding down their working life, or someone active or ‘young for their age’. Such identities are likely to ascribe particular meanings for alcohol use, behaviour which has considerable and diverse cultural connotations. In public health terms, heavy drinking and dependence on alcohol represent differing degrees of problems and health impacts. However, being a heavy drinker may underpin important community or family identities that are challenged by significant health events or bereavement in later life. Identity as someone dependent on alcohol may carry particularly adverse moral associations for older people. Relinquishing a version of self that is cherished as normal in favour of new, healthier identities implies a greater challenge in later life than simply recognising that health is at risk.

These social theories have distinct implications for understanding why older people may be drinking more. We report a qualitative study in the UK, where substantial numbers of individuals over the age of 50 exceed governmental recommendations that adults (women/men) should not drink more than 3/4 units of 8 g of alcohol per day or 14/21 units per week [11]. This research aimed to elucidate the views of older individuals aged over 50 years about alcohol consumption, health and well-being, to inform future targeted prevention in this group. The findings provide insight into older people's constructions of drinking behaviour in both health and social terms, and highlight that stigma around problem drinking in this age group may lead to moral reasoning being prioritised over public health rationales.

## Methods

This study was given favourable ethical approval by Newcastle University's Faculty of Medical Sciences Ethics Committee (application no. 000224/2009). In line with the terms of consent to which participants agreed, the data are not publicly available.

In-depth interviews (n = 24, 12 male, 12 female) were conducted between 19/11/09 and 15/03/10 by GW, a researcher with extensive experience of qualitative research on interventions for families or substance use. Purposive sampling aimed to recruit both genders and represent a broad range of age and self-reported drinking, past or current. Participants aged 50 and above were sought in order to consider views both leading up to and following the transition of retirement, and to consider how problems after the age of 65 may arise in late working life. Three local branches of a national charity aiming to improve later life (Age UK) and two regional services for alcohol problems were given research information leaflets. These explained the reasons and objectives for the research; what was involved in participation; how information would be handled and used; and who to contact for further information. The leaflets were distributed to service clients or volunteers who had experience of drinking alcohol at any level of consumption. Staff members invited the clients to consider participating in an interview, answered any questions they had about the research and asked those who were interested to complete and sign a consent form. GW contacted all potential participants by telephone to arrange a single interview; both on this call and prior to commencing interviews, the researcher checked that the individual was familiar with the research leaflet, answered any questions they had, reminded them of the voluntary and confidential nature of participation and checked that they were still happy to proceed. Initially 17 participants were recruited; although heavy drinkers were not particularly targeted in recruitment, most reported experience of dependence on alcohol. One further form was received but this participant withdrew consent without supplying a reason when contacted by GW. We sought the perspectives of older people with a range of patterns of consumption, in order to avoid over-representation of very heavy drinkers. For this reason a second wave of recruitment targeted only Age UK clients who drank but considered themselves to have had no problems with alcohol. Strategic ‘snowballing’, whereby interviewees suggested other individuals who could be invited to participate, added two further interviewees who were not using services. This increased the sample to 24, at which point the research team agreed that data saturation had been reached. Fifteen of these were recruited via Age UK, six of whom were also involved with voluntary services for mental health support. Considerable diversity was achieved (see Table 1).

**Table 1:** Interviewee characteristics.

Interviewee number	Age	Gender	From interview: self-reported drinking status/behaviour	From interview: lives with
First wave				
1	61	m	Recovering dependent drinker. Abstinent 2.5 years.	Other residents
2^a^	59	f	Recovering dependent drinker. Sensible drinker for 12 years.	Adult child; Adult child's partner; Grandchild
3^a^	56	f	Dependent drinker.	Husband; Adult child
4^a^	61	m	Dependent drinker.	Alone
5	52	m	Recovering dependent drinker. Abstinent 2 months.	Alone
6	59	m	Recovering dependent drinker. Abstinent 4 weeks.	Wife
7	57	m	Recovering dependent drinker. Abstinent 2 years.	Wife
8^a^	74	m	3 litres whisky weekly.	Alone
9	62	m	Previously 3–4 pints 3–4 nights per week. Abstinent 6 months.	Alone
10	60	m	Recovering dependent drinker. Abstinent 1 year.	Alone
11	55	f	Recovering dependent drinker. Abstinent 9 weeks.	Alone
12	51	f	Previously 3 litres cider and 2 cans beer daily. Abstinent 1 year.	HusbandTeenage children
13	68	m	Recovering dependent drinker. Abstinent 5 years.	Unknown
14^a^	58	f	Previously 2 bottles spirits per weekend. Reduced to occasional glass of wine for past 2 years.	Alone
15^a^	65	m	Previously 13 pints beer per night. Reduced to 2–3 pints per night for 1.5 years.	Alone
16^a^	52	f	Reducing dependent drinker. From bottles of spirits to 4 pints, 5 days/week	HusbandAdult children
17^a^	70	f	Bottle of wine a day. Abstinent while hospitalised only.	Other residents
Second wave				
18^a^	78	f	Occasional minimal drinker	Other residents
19^a^	83	f	Occasional minimal drinker	Other residents
20^a^	90	f	Occasional minimal drinker	Other residents
21^a^	56	m	4–5 pints twice weekly. Reduced from previous levels	Partner & sons
22^a^	59	f	1 bottle wine nightly at one stage. Reduced to 1–2 glasses some nights.	Partner
23^a^	58	f	4 vodka & tonics twice weekly.	Partner
24^a^	72	m	4 pints/night, sometimes two gin and tonics.	Wife

a Currently consuming alcohol.

Individual claims regarding stigmatised aspects of identity are likely to be qualified in the presence of peers, and recognising this influence is important to understanding how social contexts might also affect the implementation of preventive measures to address that behaviour. Focus groups with Age UK clients or volunteers were organised towards the end of data collection to allow comparison of individual accounts with socially negotiated versions of drinking in later life. Age UK staff, who also attended the groups, were asked to invite any men or women whose age made them eligible for Age UK services and who drank alcohol. One focus group was organised at each of three local branches; all those who expressed an interest attended one of these groups. The first group comprised 9 participants (1 m, 8f, ages 79–95); the second group comprised 12 participants (5 m, 7f, ages 50–85) and the third comprised 6 participants (all female, ages 51–76). To encourage participation, focus group participants were not required to disclose personal details other than age, date of birth and address; these data were gathered on consent forms. The research team prepared topic guides (available on request) to initiate discussion or return talk to the research topics. At the groups, participants were invited by the facilitator (GW) to offer views in general rather than recounting personal experience in front of others. Interviews and focus groups lasted between 40 and 150 minutes either at individual respondent's homes or the offices of Age UK or other services, as well as one interview at Newcastle University.

Interview and focus group data were audio recorded with consent, transcribed verbatim, anonymised and loaded into NVivo software. Analysis was informed by grounded theory and discursive psychology approaches. GW repeatedly read transcripts and coded for emergent themes; early analysis informed later interviews and focus groups [37]. Passages where participants accounted for drinking were examined for discourse features such as repertoires (recurring patterns of characterisation or metaphor) or contested identity claims (where descriptions of self or another are treated as questionable) [38]. Coding was refined through discussion of emerging themes amongst all researchers. Focus group data were used to triangulate findings from individual interviews; in each section below we report on findings from individual interviews, and then consider how the focus group data inform these themes. To reach a fully consistent interpretation of the data, instances diverging from the themes were considered in terms of how they might inform the interpretation. Lay reasoning, identities and stigma were considered and brought to bear during inductive analysis as theoretical constructs useful in interpreting emerging themes. We focus here on identity formation around alcohol, then on accounts of alcohol in relation to health, and finally on any influence of gender.

## Results

### Alcohol and identities – individual interviews

In the individual interviews, two distinct and contrasting ways of presenting alcohol use in later life recurred consistently: one positive, one negative. In positive terms, alcohol was treated as something that could be enjoyed by older people who consumed it minimally or occasionally. Alcohol was drunk through choice, and therefore implied control over one's actions. Drinking offered benefits of sociability and relaxation: it kept older people from losing touch, and was associated with taking things easy. Finally, drinking alcohol could be portrayed as an integral and on-going element of community, working or family life. Some or all of these characteristics were used to account for consumption as something that maintained the quality of later life. For instance, in Excerpt 1 below, a woman describes her brother's drinking as positive even though he is ‘older than me’.

***Excerpt 1*** *: … my brother and I both enjoy drink, both enjoy a social drink. My brother, even though he's a bit older than me, still has his lads' nights out where he has a damn good skinful, comes home after putting the world to rights and feels great, that can't be bad.* (Interviewee 22, female, 59 years)

The ‘skinful’ is presented as something taken with friends on occasions and through choice. It is part of a tradition connecting later life to earlier years and associated colleagues (‘still has his lads' nights out’) and has beneficial consequences (‘feels great’). By invoking these characteristics the speaker invites evaluation of this drinking behaviour as positive (‘damn good’, ‘can't be bad’).

At other times in the interviews, alcohol consumption by adults in general in later life was characterised as unacceptable or problematic. In negative terms, alcohol was used to cope with or ‘blot out’ difficulties in life. It was drunk excessively or frequently, and secretively or alone rather than in company. Consumption was driven by compulsion rather than choice and therefore implied a loss of self-control. Finally, drinking alcohol was presented as being in some way inconsistent with traditional behaviour or the way things were in earlier years. Some or all of these characteristics were invoked to present alcohol consumption in later life as negative. These sets of features, positive and negative, were always applied separately in interviews. Interviewees did not, for instance, report losing self-control on occasional nights out with former workmates; nor did they describe drinking simultaneously for enjoyment and to blot out difficulties. For example, in Excerpt 2, one man distinguishes his own drinking as positive formerly, when moderate, but negative in later years when dependent.

***Excerpt 2*** *: I guess the social element of it, going down the pub having a few beers, [yeah] talking to the guys, game of darts, or maybe it was a party or something round at someone's place and New Year's Eve or a birthday party. And it was the social involvement, you just had a few beers and [uh uh] then eventually the few beers, it got to the stage where I couldn't have a few beers, once I had a beer I always had to have something else, I needed more and more and more. [yeah] And then it switched onto wine and spirits* [liquor], *I could never have one, I always had to have more.* (Interviewee 16, female, 52 years)

He emphasises that beforehand, his drinking was a controlled choice in that he stopped at ‘a few’; it was associated with special occasions and sociable activities with peers. As such, it is consistent with the picture of late life drinking offered by the speaker in Excerpt 1, and the behaviour is presented as normal here through the use of ‘you just had…’. A separate stage of problematic drinking, in contrast, involved an escalation to more and stronger alcohol than he had drunk previously in order to cope, and a compulsion to drink which he “could never” avoid (implying that he should have done so).

Although positive and negative accounts of alcohol use were never combined, they were not always used to distinguish moderate from heavy drinking: it was observed elsewhere that positive characteristics could be applied to relatively heavy consumption, while moderate consumption could be portrayed in negative terms. For instance, one interviewee (17) indicated that she consumed a bottle of wine (9 units) a day; such consumption is well in excess of the recommended daily limit of 3 units for women. She went on to characterise her drinking in positive terms, as in Excerpt 3.

***Excerpt 3*** *: …if somebody had found me in the corner drunk that would probably shock me into maybe stopping, but that's never happened. I would never drink to the excess where I didn't know what I was doing or anything, you know. It's only (.) I keep saying it's only 12% volume which isn't very high is it?* (Interviewee 17, female, 70 years)

Here, Interviewee 17 portrays her consumption as minimal on account of the percentage of alcohol in the wine she drinks, and therefore distinct from ‘excess’. She claims self-control, stressing that she always knows what she is doing and will not be found inebriated and helpless. By applying these positive characteristics to her drinking, she invites the interviewer to view her drinking as moderate, marked by her use of ‘only’. In the same way, alcohol consumption that would be considered low-risk in public health terms could be identified as negative by an interviewee; Excerpt 4 shows an example.

***Excerpt 4*** *: Sometimes I've gone home from there and I haven't had a drink, but I've gone in and the first thing I've said to him was ‘I could just drink a glass of wine’ and he'll say ‘well why don't you?’ Well no. It makes me feel like I want to have a one, but it's never pushed me to want one. [yeah] You know, I have to have a drink because I'm relaxed and I want a one, not because I think I need a one. But sometimes I go home and I think ‘god, I could just have a glass of wine now’. You stop because I'm used to drinking in the olden days where they only drank on a Friday and Saturday.* (Interviewee 23, female, 58 years)

The woman speaking here presents alcohol consumption as something one has to stop because it might be undertaken from necessity rather than choice, and would be inconsistent with traditional times for drinking. To the extent that she does not suggest her companion will be drinking as well, it is portrayed as an individual rather than social undertaking. The consumption to which she refers - a glass of wine at home ‘sometimes’ - represents low-risk consumption according to recommended guidelines; however, her characterisation of it is consistent with the negative, unacceptable version of alcohol.

To summarise, individual interviewees used different characteristics to label drinking in later life as either normal and thus positive, or else as unacceptable or problematic, and therefore negative. These positive and negative labels were not, however, clearly associated with distinct levels of moderate or excessive alcohol consumption.

### Alcohol and identities – focus groups

The same positive and negative features were also apparent in talk at focus groups. However, disparities between individual norms revealed the negotiation of social norms around alcohol. The flexibility in distinction between moderate and heavy drinking meant that identification of a pattern of drinking by one participant as either positive or negative could be challenged by another. ‘Alcoholics’ in general were often disparaged by the focus groups as people to be avoided or pitied, establishing the undesirability of being considered an alcoholic. Given this, negotiations took place to resolve what could be deemed positive or negative. Where claims of normality were challenged, the repertoires functioned to claim or ascribe particular identities as a positive or negative older drinker. Excerpt 5 shows an instance of this.

***Excerpt 5*** *:*

*F3: Well that's what we do if any of the family come down to visit. We go for a lunch. We go for a lunch and have like half a lager.*

*F8: You don't need to drink do you?*

*F3: You don't need to drink, but I might get one with my meal, you know. It's not a case of going out drinking. We're there for the company and the meal, so (.) that's what we do.*

*F8: Well they didn't used to eat in pubs at all.*

(Focus Group 1)

The consumption under discussion – half a pint of lager with an occasional meal – is low-risk according to UK guidelines. Participant F3 identifies this moderate drinking as normal (something ‘we do’) by presenting it as a choice within a sociable family context. That this represents an identity claim is demonstrated by participant F8's implication in response that F3 had ‘needed’ to visit a drinking venue rather than choosing to do so. By suggesting the drinking is driven by compulsion, she invites it to be seen in negative terms. Faced with this, F3 counters by restating the voluntary or occasional nature of her drinking (‘I might’) and asserting its essentially social function (‘there for the company’). F8 responds to suggest that F3's behaviour is not associated with tradition, again inviting a negative interpretation.

In a similar way, one participant's claim for the normality of heavy drinking could be challenged; Excerpt 6 shows an example of this.

***Excerpt 6*** *:*

*M3: I was away last week, and one particular gadgy* [man], *he supped 10 pints and he was immune to it, you know. I mean//*

*F4://Was he rational in everything, and could he walk?*

*M3: Well aye, you wouldn't think he'd been out.*

*F4: Had he got his senses? (.)*

*M3: He's done it all his life.*

*F4: Had he got his senses?*

*M3: He seemed to be alright to me like. Or not me [laughs], I had about 8, but you know this gadgy he'd done it all his life, and nowadays, it's now the young ones that get 10, they'll go and put shop windows in, because they can't take it.*

(Focus Group 2 – ‘M’ denotes male participant)

Participant M3 presents the drinking of 10 pints by a drinking companion in one session as controlled and occasional. F4, however, questions whether the drinker in question could have been ‘immune’ to the effects of this consumption (considerably in excess of recommended daily guidelines) and implies loss of self-control of this person's reason and ability to walk. M3 rebuts this, but faced with a repeated challenge he resists the insinuation of negative drinking by asserting that his drinking companion had life experience within which this was normal. This is finally reinforced by aligning his own drinking with that of his companion and treating this as humorous, then suggesting that only younger people without their experience are likely to lose self-control after such consumption.

Exchanges such as these show that not only are definitions of moderate and heavy drinking flexible, they are contested among older adults in establishing the parameters of acceptable behaviour. To the extent that self-control and adherence to traditional practices were treated as key characteristics of normal drinking, propriety emerged as central for participants in evaluating levels of alcohol consumption.

### Health and changing drinking behaviour – individual interviews

Individual interviewees did not refer spontaneously to health when discussing alcohol consumption; however, the researcher specifically asked about health as a topic of research interest in the individual interview schedule. Analysis explored how participants treated their health as a factor when accounting for choosing either to drink less, or not to do so.

In the individual interviews, some interviewees reported a decision to reduce or abstain from alcohol consumption. They referred to a range of chronic conditions, and the use of medicines prescribed for these, in relation to these decisions. Narratives of a major injury or health scare, such as hospitalisation for pancreatitis or pneumonia were commonly concluded with a decision to cut down. Some conditions or events such as falls were explicitly attributed to drinking, typically where the speaker recognised an impact of alcohol on their symptoms; the experience of symptoms getting worse with drinking could be offered to rationalise the need to cut down. For instance, the speaker in Excerpt 7 attributes his tendency to fall at least partly to drinking.

***Excerpt 7*** *: I still haven't packed in smoking, but I've packed in drinking because of the balance. As you know, if I have too much to drink I just go backwards.* (Interviewee 9, male, 62 years)

This was particularly, but not solely, the case with those who identified themselves as formerly dependent on alcohol. The experience of side-effects from drinking while on medication could also be used to explain cutting down, for instance by a woman on medication for bipolar disorder who described ‘terrible hangovers’ when she had also drunk alcohol. Those saying they had reduced drinking for health reasons pointed to resulting improvements in their symptoms.

Other interviewees described continuing to drink heavily despite experiencing impaired health. This could be accounted for as self-medication to relieve symptoms, as in Excerpt 8.

***Excerpt 8*** *: The pain. Even some of my bones with my osteoporosis. My ankles, my everything. The pain and all that. As soon as I start having a few pints it goes away.* (Interviewee 4, male, 61 years)

Where interviewees identified themselves as dependent, some explained that they were unable or that it was ‘too late’ to change their drinking regardless of whether it had an impact on other health conditions. In particular, they described a cycle of mental health and alcohol problems that kept them using alcohol: drinking exacerbated their conditions in its aftermath, but going without alcohol meant facing a return of feelings or symptoms, as in Excerpt 9.

***Excerpt 9*** *: I've told the doctor there's no way I'll stop drinking. There's no way I can stop drinking. There's too much up there, and it's too late. Too much in my head.* (Interviewee 3, female, 56 years)

Elsewhere in the interviews, health conditions in later life were described as unrelated to heavy drinking, either because overall health and wellbeing was relatively good for their age and symptoms were not experienced, or because symptoms were perceived as being unaffected by drinking. For instance, in Excerpt 10, a man who had suffered a stroke discounts health professionals' advice to reduce consumption since he feels in relatively robust health.

***Excerpt 10*** *: I'm 55, I'm perfectly fit, I don't run about beating everyone up, I'm not incontinent, I'm perfectly healthy, I get a check for my stroke, which coincidentally, they check my bloods, they check my liver, check my pancreas, check my kidneys you know, everything's fine, so how is this half a dozen pints doing any irreparable damage?* (Interviewee 21, male, 56 years)

Interviewees who described drinking while taking prescribed medication also said they judged any threat to their health from this according to whether they felt ‘fine’. Health risks were also discounted on various grounds that medications would be ‘in my system’ before alcohol mixed with them (as in Excerpt 11) or would not have been prescribed if unsafe to take with alcohol.

***Excerpt 11*** *: I still take them* [tablets] *anyway and I've been taking them regular. It hasn't done nowt* [nothing] *wrong to me but numbing my brain a bit. That's all I can see what's happening. If that was the case I would just leave the tablet alone and just stop on the beer (.) I take them on a morning anyhow, and they're probably in my system by the time I have drink and stuff like that.* (Interviewee 4, male, 61 years)

Choosing to drink heavily while suffering health conditions could be perceived as irrational. When reporting this behaviour, interviewees' talk anticipated this: for instance, Excerpts 10 and 11 are framed to question or refute such a perception (‘how is this … doing any damage?’, ‘hasn't done nowt wrong to me’). Links between alcohol intake and poor health were also more explicitly challenged; some participants described themselves as having disproved doctors' warnings about alcohol affecting their health. UK government guidelines for low-risk drinking were usually referred to sceptically for being unrealistic or failing to take account of individual capacities; Excerpt 12 gives an example of this.

***Excerpt 12*** *: Now, I'm older, my body is bigger than yours so I could obviously take more drink than you. So people aren't stupid, they know that people take drink in different ways. So making it just a huge umbrella and saying ‘you drink 4 units and you'll be alright’, it doesn't make sense.* (Interviewee 23, female, 58 years)

### Alcohol, health and changing drinking behaviours – focus groups

Like individual interviewees, focus group participants did not refer spontaneously to health when discussing alcohol consumption. In response to questions about health, it was again suggested that experiencing a physical impact from health conditions or impairments warranted reduced alcohol use as part of a generally healthier lifestyle. For instance, in Excerpt 13, a participant asserted that drinking less was consistent with being a cancer survivor, without attributing that illness to alcohol use per se.

***Excerpt 13*** *:*

*F2 …I've had a serious cancer operation which you all know, but I don't drink; I have a drink, I drink, not a lot of [it]; I have a couple of lagers or something: finished. I don't go over the top, but that's individual. You know that you've been, you've been very ill and this sort of thing and it's no good pushing it down your neck, because you're only making yourself worse…*

(Focus Group 2 – ‘F’ denotes female participant)

Continuing to drink heavily while suffering a health condition or taking medication, however, was only attributed to ‘other’ older people rather than those present. In this respect, there appeared to be tacit recognition that heavy drinking with a chronic condition should be seen as a health concern. However at points in two of the groups, participants reached a consensus regarding scepticism about medical advice provided on alcohol. It could be agreed, for instance, that health professionals were overly ready to attribute older people's health problems to their alcohol intake, as in Excerpt 14.

***Excerpt 14*** *:*

*F3: Even, even if you go to an asthma clinic they ask you if you drink and how many units in a week.*

*F5: And really what has it got to do with anything?*

(Focus Group 3)

Another basis for agreement was a belief that evidence on health risks from alcohol was contradictory or changeable and therefore inconclusive; Excerpt 15 gives an example of this.

***Excerpt 15*** *:*

*F1: I don't think that anybody has the authority//*

*F2://No, I don't think they have got the right//*

*F1://or should in any way, shape or form stop anybody from drinking if they want to.*

*F4: Because it's bad for your health.*

*F2: But everything is bad for your health.*

*F1: Exactly.*

*F2: I mean eggs, you cannot have eggs, or you couldn't.*

*F1: Well, every other day there's something they tell you is bad for you.*

(Focus Group 3)

In this exchange, participants agree that people should not be prevented from drinking on health grounds because too many products or behaviours have, or could be, found to carry health risks. Medical practitioners' encouragement of reduced drinking can be branded unreasonable if driven by health warnings from research that are excessive: if ‘everything is bad for you’, then it is irrational to assume that health risks can be avoided. The example of revised warnings against eating eggs is cited to emphasise that such public health messages can be regarded as provisional pending future studies.

### Gendered patterns of drinking

Individual interviewees rarely made links between gender and drinking behaviour. However, some subtle distinctions were made by participants regarding culturally appropriate drinking practices among men and women in this setting. For example, men's heavy consumption of beer with groups of other men in pubs was valorised, as in Excerpt 16, while women drinking beer or going to pubs, particularly if alone, would be viewed negatively, as in Excerpt 17:

***Excerpt 16*** *: …now I've sat with them and I've drunk with them. If they drink 15 pints a night or drink until they fall down, that makes them a man. (Interviewee 15, male, 65 years)*

***Excerpt 17*** *:*

*Interviewee: But since he [husband] died 18 years ago I've never been in a pub since*

*GW: No? Why did you stop going?*

*Interviewee: Well when you're on your own you don't. (Interviewee 12, female, 70 years)*

However, such attitudes or behaviours were often presented as a feature of past behaviour. Some participants who had experienced alcohol dependence described drinking in a way that challenged traditionally gendered patterns: for instance, a man who had tended to drink sweet wine at home, or women who described drinking beer in pubs or with male friends. Rather than separately gendered patterns of consumption, couples drinking together emerged as a characteristic of retirement compared with earlier life. There were however some indications that men could regret this shift away from earlier drinking with peers, as in Excerpt 18.

***Excerpt 18*** *: …one or two of them [lads], because they're 50 odd years, the same age, they're taking more the role of the husband taking the wife out. I take the wife out, but the Friday night, I think, is sacrosanct. But now it's getting less and less so.* (Interviewee 21, male, 56 years)

Focus group accounts were also almost always voiced in terms of ‘older people’ or people in general. The few gender-specific aspects of drinking brought up were usually references to men or women typically drinking together in groups on a regular ‘lads’ or ‘girls’ session, as in Excerpt 19.

***Excerpt 19*** *:*

*F4: my husband and a lot of the lads up here work with the shipyards; they all meet in one of the clubs over the water on a Saturday afternoon, and there's still about 15 of them all get over there, and they have a drink.*

(Focus group 2)

For men, this meant drinking together in pubs, typically after work to ‘get in amongst the fellas’ as one participant put it. Such drinking was emphasised at the groups as positive, intended for socialising and not getting drunk. This is in contrast with individual interviewees in Excerpts 2 and 16 above, who depicted heavy drinking with ‘the lads’ in pubs as a precursor to their dependence on alcohol. At the all-female focus group, participants anticipated that men should drink beer in pints, with one participant in Excerpt 20 finding it ‘unmanly’ for her partner to drink otherwise:

***Excerpt 20*** *:*

*F5: Sometimes I could clip [partner's name] because he'll always get a half pint and I want him to have a pint. [laughs]*

*F4: Good for him*

*F5: Because I don't think it looks macho with this stupid little half pint.*

(Focus Group 3)

Female participants in the focus groups also identified that women drinking in pubs could be seen in negative terms. However, any differences in how men and women drank were typically proposed in relation to an earlier stage in life or days gone by, with the speaker or another participant suggesting that this was no longer the case, as in Excerpt 21.

***Excerpt 21*** *:*

*F2: I think a lot of the men go out a lot more. The older men do still drink. Because that's their only entertainment really, isn't it? I would say middle aged men -*

*F5: I don't think it's just men. I think ladies can drink just as much as men.*

*F2: I'm talking about going out for a drink though, you know. Do you think the older ladies do?*

*F5: Oh yeah, I would say so*

(Focus Group 2)

Whilst specific references to gender were infrequent, the fact that they were evident at all suggests that older adults recognised gender-based patterns of drinking and also issues of stigma in relation to public drinking by women and deviations from male drinking stereotypes, which could challenge attempts to reduce consumption, such as drinking smaller quantities of alcohol.

## Discussion

The aim of this research was to contribute to contemporary understandings of alcohol use in later life and its implications for the health of older people. UK adults in later life who were asked to talk about alcohol and health oriented strongly towards the opposed identities of normal or problematic drinker, the latter being highly stigmatised. These identities were defined primarily by self-control and propriety, rather than health considerations. In data from focus groups, participants could be seen to negotiate consensual views on alcohol and its implications for both health and propriety, providing support for their views being socially constructed in response to a particular context. These older people portrayed drinking less alcohol as an appropriate response if one experienced impaired health. However, continued heavy drinking could be presented as normal behaviour for someone experiencing relative wellbeing in later life, or if their experience of ill health was construed as unrelated to alcohol consumption. This normalisation was supported by treating general health advice on alcohol as questionable or individually irrelevant. Drinking patterns did not appear to be strongly defined by gender, although some gendered expectations of drinking were evident.

Absolute numbers of risky drinkers in older age groups are small compared with younger adults but will grow in an ageing population [15], [39]. It is predicted that 4.4 million US adults aged 50 or above will need treatment for substance use in 2020 compared with 1.7 million in 2000 [39]. More stringent guidelines on alcohol consumption for those aged 65 and over are often recommended by governments but not always formally instituted [15], [40]. There is a need to develop preventive approaches that specifically target older populations, for instance providing longer term in-home support, tailored information on risks to health from alcohol use in later life, or health workers with specific training on older people's needs in relation to alcohol [41]. Non-specialist preventive approaches to avoid the harmful consequences of continued heavy drinking, such as brief interventions, are effective with older people [42], [43]. Nevertheless, preventive measures rely on reasoning in terms of health risks and benefits, and so it is notable that health risks from drinking, as opposed to health impacts, did not appear meaningful to our participants.

The scepticism displayed by participants towards health advice regarding alcohol consumption is consistent with resistance to health promotion reported for other lifestyle issues, such as smoking [44], [45]. Advice on risks from alcohol must compete for older people's attention with many other warnings and may be less meaningful because of a general perception of ‘overkill’ [46]. Focus groups facilitate qualification of one participant's views by others and so represent a particularly fertile environment for such reasoning to emerge, for instance in making reference to famous instances of untrustworthy advice [47]. Crossley [48] found a similar recollection of unhelpful UK government advice about salmonella risk from eggs to that raised by one of our focus groups. However, individual reasoning may not be consistent with socially negotiated points of view. Participants' questioning of advice on alcohol when they had not experienced its adverse impact on their health was characteristic of lay understandings, which are crucially informed by lived experience or personal observation and the desirability of the health behaviour [24], [49]. Their reasoning appeared to conform to public health rationales where health risks were no longer perceived as deferred [cf 23]; thus behaviour change was warranted only within a narrative of damaged health, or once harmful or dependent drinking identity was recognised. Yet participants described experiencing adverse health and increased need for treatment and care in later life; in this respect older people might be expected to be more likely than younger populations to consider issues in health terms.

Increasing alcohol consumption among older age groups might instead reflect identity work in this population. Older adults can be relatively ill-informed about alcohol measurements on which guidelines are based [50], [51]. Participants reasoned that recommended guidelines are inconsistent with individual capacities, but also showed concern that being considered an unhealthy drinker, even at low levels of drinking, might signify alcoholism. Given that constellations of positive and negative aspects were not consistently applied by individuals to discrete levels of moderate and heavy drinking, they can be viewed as repertoires [52]: self-contained sets of characteristics used to associate an individual's drinking with a particular identity, whatever they actually consume. They characterise positive or negative identities rather than objectively distinct behaviours. The positive repertoire invites the idea of a normal and therefore acceptable drinker; the negative repertoire identifies an individual's drinking as a source of stigma. Interviewees saying they drank showed strong sensitivity to being perceived as the ‘wrong’ sort of drinker (irresponsible, improper, or wilfully abusing alcohol) and those identifying themselves as previously dependent on alcohol treated this as a negative aspect of their former selves.

Drinking behaviour did not appear to be strongly defined by gender in our study in contrast to other qualitative work. This difference may reflect the fact that gender-based issues were not the main focus of this study. Some gendered expectations of drinking were described, often with a focus on culturally stereotyped patterns of consumption. In line with findings from somewhat younger UK men [53], both men and women treated regular drinking of ‘pints’ or socialising in ‘pubs’ together as normal for men but not women. In the present study, however, gendered drinking patterns tended to be presented as more widely adhered to in earlier life. Furthermore, individuals who had experienced alcohol dependence described overruling those gendered patterns that were identified. In addition, it was noted that drinking could become less gender segregated in later life, as some couples spent more time together. Further qualitative research focused on later life drinking in males and females is needed to unravel these complex social and health issues.

Longitudinal studies of large samples of older adults in both the US and Norway have shown reduced drinking linked to the incidence of chronic or painful medical conditions [5], [54] or acute health events [20]. Such changes in wellbeing may provide a narrative framework to account for reducing one's drinking and may constitute opportunities for engaging the interests and attentions of those who drink to excess [55]. Preventive approaches should aim to target unhealthy behaviour before it impairs health; yet older adults who feel well appear to question longer term health risks from heavy drinking, or feel that the risks are outweighed by benefits of sociability and relaxation. The talk of older adults indicates that unhealthy drinking may not imply a spoiled identity [28], and therefore represent a cause for concern, unless it can be read by others as a loss of self-control. However, acknowledging that one's health might be affected by alcohol implies a loss of control over physical integrity and therefore risks incurring undesirable identity as an ‘alcoholic’. In this way, discourse practices reinforce or maintain stigma but can also have an important role in re-constructing self-identities [55]. To counter heavy drinking in older populations before it has significant impacts on health, interventions must engage and foster positive identities for older adults that can support change. Social marketing approaches would be appropriate for this and have been used to promote healthier lifestyles in this age group [56]. In particular interventions should aim to reframe the widely recognised but poorly understood concept of alcoholism. For instance, the information component of brief advice could be tailored to clarify the mechanism of cumulative impacts on health through drinking at levels short of dependence over a number of years, and how and why such impacts are strengthened in later life.

Although the overuse of alcohol in later life and its associated stigmas are clearly concerns for public health [15], [18], [30], [57] these issues have not been adequately explored through the framework of social theories to date. Qualitative methods have allowed this study to access social constructions grounded in older peoples' own frames of reference. It is a limitation that recruitment was mostly through one agency, and a larger study might have adopted a broader strategy. Nevertheless, the sample was diverse and from a UK region with an older population than average and a culture of heavy drinking [58], [59]. Stigma around drinking in later life has been observed in other developed countries besides the UK, and discourse observed here around propriety and health is likely to be analogous to that in other locations and populations. For instance, working adults in mid-life in Glasgow and the North East also identify a tradition of nights out drinking with colleagues [32], [60], though those studies found somewhat more readiness, particularly among men, to endorse inebriation as a goal than among the older adults in the present study. Although larger than many estimates of numbers needed to reach data saturation [61], the size of sample in this study is appropriate given the variation encompassed. Participants reported varied consumption and ages ranged from 50–90. As such, findings extend those based on treatment populations only [62]. The interview sample included a greater proportion of individuals identifying themselves as currently or formerly dependent on alcohol than might be expected in the overall population of older people. However individual views were triangulated with those from the focus groups, where a normative identity as a sensible drinker was adhered to by all participants. The ratio of women to men within the groups (21∶6) was considerably greater than in the UK population aged 65 and over (around 5∶4) [63]. This may reflect a greater willingness among older women compared with older men to participate in group discussions. However, the unit of analysis for focus group data should be considered the group given that not all participants may contribute or contribute equally [64], and two of the three groups were mixed.

Consideration of our data in light of theories of lay epidemiology and identities has informed an under-recognised area of public health concern. Strengthening the evidence for specific risks to health from alcohol and clarifying how these interact with behaviour might increase the impact of preventive strategies to reduce heavy drinking in later life. However, the processes by which identities can be constructed are ‘tremendously powerful’ in governing social interaction ([38], p5), and the concept of identities can also improve understanding of public health issues around older people's alcohol-related behaviour. Although older people construct positive identities for themselves by discounting stigma [65], emphasising propriety to avoid stigma around alcohol could lead them to treat unhealthy behaviour as normal, such as the drinking of eight pints of beer endorsed in Excerpt 6 on the grounds of apparent self-control. Long-term comparative data indicate that consuming alcohol at high risk in later life tends to reinforce some social resources at the expense of others [66]; older men's heavy drinking is positively associated with friends' approval for drinking, but is likely to be reduced over time if they have a partner who drinks less heavily [67]. Access to a strong identity as a moderate drinker might enable older adults to resist normative influences within relationships that support heavy drinking. This identity should retain strengths such as sociability without implying susceptibility to alcoholism. Community-level interventions to reinforce heavy drinking as unhealthy but not necessarily synonymous with dependency would facilitate older people's discussion of alcohol use with services, particularly if they clarified specifically how and why health can be at risk from heavy drinking in the absence of immediate symptoms. Future research could extend our findings by exploring identities around such interventions, and among older adults not using services.
